# The American Academy of Dental Science

**Published:** 1883-01

**Authors:** H. F. Hamilton

**Affiliations:** 124 Commonwealth Ave., Boston


### PROCEEDINGS.

### The American Academy of Dental Science.

The fifteenth annual meeting of the American Academy of Dental Science was held at Young's Hotel, Boston, October 25, 1882. The morning session was chiefly devoted to the regular business, and to the election of the following-named officers: President, Dr. G. T. Moffatt; vice president, Dr. J. H. Batcheller; recording secretary, Dr. H. F. Hamilton; corresponding secretary, Dr. E. B. Hitchcock; treasurer, Dr. E. H. Smith; librarian, Dr. H. C. Meriam; executive committee, Dr. C. P. Wilson, Dr. E. C. Briggs and Dr. J. S. Mason. Dr. S. J. Shaw and Dr. F. E. Banfield were elected active fellows. The afternoon session opened with the annual address by Dr. Frank Abbot, of New York, on "Dental Education." The paper was chiefly devoted to emphasizing the necessity of young men's having a thorough medical education as a preliminary to the study of dentistry, and the need of Massachusetts following the other States in obliging practicing dentists to have a suitable degree, or to pass an examination before a State examining board. Dr. W. H. Atkinson, of New York, read a paper on "Artificial Teeth," giving valuable ideas on the subject, and on the healing of tissues. He unqualifiedly condemned celluloid and vulcanite as base-plates. Dr. A. W. Buckland, of Woonsocket, R. I., read a paper describing a method of filling by covering a lining of cement with amalgam while both are in a plastic state. He deprecates the use of gold and severe methods with patients needing gentle treatment. Resolutions were passed on the death of Dr. J. E, Fiske, of Salem, and Dr. W. H. Allen, of New York, both honorary fellows of the academy. The annnal dinner took place at five o'clock. Many distinguished dentists from other cities were present, and the meeting was one of the most valuable, both professionally and socially, ever held.

H. F. Hamilton,
124 Commonwealth Ave., Boston. Recording Secretary.

